# Properties, quantile regression, and application of bounded exponentiated Weibull distribution to COVID-19 data of mortality and survival rates

**DOI:** 10.1038/s41598-024-65057-6

**Published:** 2024-06-21

**Authors:** Shakila Bashir, Bushra Masood, Laila A. Al-Essa, Aamir Sanaullah, Iram Saleem

**Affiliations:** 1 Department of Statistics, Forman Christian College (A Chartered University), Lahore, Pakistan; 2 Department of Mathematical Sciences, College of Science, Princess Nourah bint Abdulrahman University, P.O.Box 84428, 11671 Riyadh, Saudi Arabia; 3 Department of Statistics, COMSATS University Islamabad, Lahore Campus, Lahore, Pakistan

**Keywords:** Computational science, Scientific data, Statistics

## Abstract

Well-known continuous distributions such as Beta and Kumaraswamy distribution are useful for modeling the datasets which are based on unit interval [0,1]. But every distribution is not always useful for all types of data sets, rather it depends on the shapes of data as well. In this research, a three-parameter new distribution named bounded exponentiated Weibull (BEW) distribution is defined to model the data set with the support of unit interval [0,1]. Some fundamental distributional properties for the BEW distribution have been investigated. For modeling dependence between measures in a dataset, a bivariate extension of the BEW distribution is developed, and graphical shapes for the bivariate BEW distribution have been shown. Several estimation methods have been discussed to estimate the parameters of the BEW distribution and to check the performance of the estimator, a Monte Carlo simulation study has been done. Afterward, the applications of the BEW distribution are illustrated using COVID-19 data sets. The proposed distribution shows a better fit than many well-known distributions. Lastly, a quantile regression model from bounded exponentiated Weibull distribution is developed, and its graphical shapes for the probability density function (PDF) and hazard function have been shown.

## Introduction

The need to develop the unit interval distributions due to their applications in engineering, economics, psychology, and biology are quickly increasing. The unit interval or distribution bounded with the interval [0,1] are significant for modeling data given in the intervals between zero and one, such as ratios, rates, and percentages. For example, in psychology the percentages and proportions are useful to judgement possibilities, the percentage of mind section captured by a specific region. In economics, under study variable or data is generally limited to unit intervals e.g., market share, capital structure, and percentage of income spent on non-permanent utilizations. It is also observed that unit distributions have attractive hazard rate shapes like a bathtub. A well-known distribution named two parameter beta distribution is bounded with data between zero and one but due its intractable cumulative distribution function (CDF) and quantile function (QF), beta distribution has some limitations. The procedure of generating random observations from the beta distribution is difficult due to the complexity of the expressions of its QF and CDF. Therefore, researchers were inspired to develop such type of unit interval distributions having attractive expressions for CDF, QF and variety of shapes for the hazard functions.

Several noteworthy contributions can be found in the literature, such as the unit inverse Gaussian distribution (UIGD) introduced by^1^ and the unit Lindley distribution (ULD) proposed by^2^. Additionally, a multivariate quasi-beta regression model for bounded data developed by^3^, while the unit generalized half normal distribution generated by^4^. The unit gamma/Gompertz distribution introduced by^5^, and a quantile regression model based on the unit Birnbaum-Saunders distribution have produced by^6^, demonstrating its applications in medicine and politics. Furthermore, the transmuted unit Rayleigh quantile regression model developed by^7^, adding to the repertoire of methodologies available for analyzing unit interval datasets. The continuous distribution with support on the unit interval continues to advance as researchers strive to address the challenges associated with modeling and analyzing such data. By developing new bounded distributions and innovative regression models, scholars are expanding the toolkit available for studying unit interval datasets across various disciplines. These advancements are expected to have a profound impact on the fields of engineering, economics, psychology, and biology, enabling more accurate and effective analysis of data involving ratios, rates, and percentages within the unit interval [0, 1]. Nasiru et al.^8^ introduced a new lifetime distribution named as bounded truncated Cauchy power exponential distribution to model the unit data, Almazah et al.^9^ executed various distribution methods to find five new different forms of the inverse Weibull model and the resultant models applied on the mortality rate of COVID-19. Moraes-Rego^10^ introduced a unit interval distribution named a truncated exponentiated exponential distribution. Afify et al.^11^ developed and applied discrete exponential distribution to COVID-19 data. Maya et al.^12^ introduced bounded probability distribution and named it unit distribution, they derived its various properties and finally applied to the real data sets. Mustafa & Zehra^13^ developed the unit log–log distribution, with quantile regression modelling and applied to educational measurements. Hannan et al.^14^ introduced unit exponential Pareto distribution with properties and modeled it on the recovery rate of COVID-19. Ayuyuen & Bodhisuwan^15^ developed the unit Garima distribution with properties. Sangsanit & Bodhisuwan^16^ introduced Topp-Leone generator of distributions.

This study addresses the limitations of existing unit interval distributions by proposing a novel solution, the bounded exponentiated Weibull (BEW) distribution. The proposed BEW distribution fills a crucial gap in the existing literature by providing a dedicated distribution specifically designed for unit interval data. This fills an important need in fields such as reliability analysis, survival analysis, and time-to-event modeling, where unit interval data frequently arise. By addressing this gap, the paper contributes to the advancement of mathematical methodologies in these areas. Therefore, unlike other distributions, the BEW distribution offers an attractive cumulative distribution function and quantile function, making it better suited for describing various types of datasets within unit intervals. The paper also explores the influence of the widely used Weibull distribution in inspiring the development of the BEW distribution. Additionally, the paper introduces a bivariate extension of the BEW distribution to model the independence among random data over unit intervals. Lastly, the paper presents a novel quantile regression model based on the BEW distribution, enabling the investigation of the relationship between a given covariate and a response variable. By employing BEW distribution-based quantile regression, researchers can enhance their mathematical modeling techniques and broaden their applications across domains such as economics, social sciences, and finance. This novel approach has the potential to accelerate research in these areas by providing new insights into the relationships between variables.

## Methods

This section presents a methodology for developing a bounded exponentiated Weibull (BEW) distribution along with its properties.

### New proposed BEW distribution

Consider, a CDF and a probability density function (PDF) of an exponentiated Weibull distribution respectively are given by,

1 $${\mathbf{F}}\left( {\mathbf{y}} \right) = 1 - \left[ {1 - {\varvec{e}}^{{ - \left( {{\mathbf{\lambda y}}} \right)^{{\varvec{\beta}}} }} } \right]^{{\varvec{\alpha}}} ,\user2{ }{{\varvec{\uplambda}}}, {\varvec{\alpha}},{{\varvec{\upbeta}}} > 0 \& {\mathbf{y}} > 0$$

2 $${\text{and}}\;\;{\mathbf{f}}\left( {\mathbf{y}} \right) = {\varvec{\alpha}}{\mathbf{\lambda \beta }}{\varvec{e}}^{{ - \left( {{\mathbf{\lambda y}}} \right)^{{\varvec{\beta}}} }} \left[ {1 - {\varvec{e}}^{{ - \left( {{\mathbf{\lambda y}}} \right)^{{\varvec{\beta}}} }} } \right]^{{{\varvec{\alpha}} - 1}} ,\user2{ }{{\varvec{\uplambda}}}, {\varvec{\alpha}},{{\varvec{\upbeta}}} > 0 \& {\mathbf{y}} > 0$$

Now, a distribution termed as the BEWD is developed following the conversion of $e^{ - X} = {\text{Y}} \to - {\text{ln}}\left( {\text{Y}} \right) = {\text{X}}$. The CDF of the BEWD is as follows,

$$F_{Y} \left( {y;{ }\lambda ,\alpha ,\beta } \right) = {\text{ P}}\left( {e^{ - X} \le y} \right){ ,}$$

$$F_{Y} \left( {y;{ }\lambda ,\alpha ,\beta } \right) = {\text{ P}}\left( { - {\text{X}} \le {\text{ln}}\left( {\text{Y}} \right)} \right),$$

$$F_{Y} \left( {y;{ }\lambda ,\alpha ,\beta } \right) = {\text{ P}}\left( {{\text{X}} \le - {\text{ln}}\left( {\text{Y}} \right)} \right),$$

or

$${ }F_{Y} \left( {y;{{ \lambda }},\alpha ,\beta } \right) = 1 - { }F_{X} \left( { - \ln \left( {\text{Y}} \right);{{ \lambda }},\alpha } \right).$$

Now, the following is a CDF of the BEW distribution,

3 $${\mathbf{F}}\left( {\mathbf{Y}} \right) = 1 - \left[ {1 - {{\varvec{\Upsilon}}}} \right]^{{\varvec{\alpha}}} , {{\varvec{\uplambda}}}, {\varvec{\alpha}},{{\varvec{\upbeta}}} > 0 \& 0 < {\mathbf{y}} < 1,$$

where ${\varvec{y}}^{{{\varvec{\uplambda}}}} = {{\varvec{\Upsilon}}},$ ${{\varvec{\uplambda}}}$ is a location parameter, while ${\varvec{\alpha}}$ & ${{\varvec{\upbeta}}}$ are respectively shape and scale parameters.

Hence, the PDF of the BEWD is given by

4 $${\mathbf{f}}\left( {\mathbf{y}} \right) = {{\varvec{\uplambda}}}{\varvec{\alpha}}{{\varvec{\upbeta}}} {{\varvec{\Upsilon}}}{\varvec{y}}^{ - 1} \left[ {1 - {{\varvec{\Upsilon}}}^{{\varvec{\beta}}} } \right]^{{{\varvec{\alpha}} - 1}} \left( {{\varvec{\Upsilon}}} \right)^{{{\varvec{\beta}} - 1}} , {{\varvec{\uplambda}}}, {\varvec{\alpha}},{{\varvec{\upbeta}}}, > 0, \& 0 < {\mathbf{y}} < 1,$$

### Reliability measures of BEW distribution

In this section a few reliability measures such as survival function, hazard function, reversed hazard function, cumulative hazard function, odd function, elasticity, and mills ratio for the BEW distribution have been discussed.

The survival function represents the probability that an individual will survive beyond a certain time, denoted as y. In the case of the BEW distribution, the survival function can be expressed as,

$${\mathbf{S}}\left( {\mathbf{y}} \right) = \left[ {1 - {{\varvec{\Upsilon}}}^{{\varvec{\beta}}} } \right]^{{\varvec{\alpha}}}$$

On the other hand, the hazard function characterizes the death rate of an individual at a specific age, denoted as y For the BEW distribution, the hazard function can be calculated as,

5 $${\mathbf{h}}\left( {\mathbf{y}} \right) = \frac{{{\varvec{f}}\left( {\varvec{y}} \right)}}{{1 - {\varvec{F}}\left( {\varvec{y}} \right)}}$$

6 $${\mathbf{h}}\left( {\mathbf{y}} \right) = \frac{{{{\varvec{\uplambda}}}{\varvec{\alpha}}{{\varvec{\upbeta}}} \left[ {1 - {{\varvec{\Upsilon}}}^{{\varvec{\beta}}} } \right]^{{{\varvec{\alpha}} - 1}} {{\varvec{\Upsilon}}}^{{\varvec{\beta}}} }}{{ \left[ {1 - {{\varvec{\Upsilon}}}^{{\varvec{\beta}}} } \right]^{{\varvec{\alpha}}} {\varvec{y}}}}$$

Additionally, the reverse hazard function determines the fraction of the life probability density to its distribution function. In the case of the BEW distribution, the reverse hazard function can be defined as, ${\varvec{r}}_{{\varvec{h}}} \left( {\varvec{y}} \right) = \frac{{\user2{F^{\prime}}\left( {\varvec{y}} \right)}}{{{\varvec{F}}\left( {\varvec{y}} \right)}}$ = $\frac{{{\varvec{f}}\left( {\varvec{y}} \right)}}{{{\varvec{F}}\left( {\varvec{y}} \right)}},$

7 $${\varvec{r}}_{{\varvec{h}}} \left( {\varvec{y}} \right) = \frac{{{{\varvec{\uplambda}}}{\varvec{\alpha}}{{\varvec{\upbeta}}}\user2{ }\left[ {1 - {{\varvec{\Upsilon}}}^{{\varvec{\beta}}} } \right]^{{{\varvec{\alpha}} - 1}} \user2{ }{{\varvec{\Upsilon}}}^{{\varvec{\beta}}} }}{{\left[ {1 - \left[ {1 - {{\varvec{\Upsilon}}}^{{\varvec{\beta}}} } \right]^{{\varvec{\alpha}}} } \right]\user2{ y}}}.$$

The cumulative hazard function for the BEW distribution is

8 $$H\left( x \right) = - \alpha ln\left[ {1 - {\Upsilon }^{\beta } } \right]$$

The odd function for the BEW distribution is

9 $$O\left( t \right) = \frac{1}{{\left[ {1 - {\Upsilon }^{\beta } } \right]^{\alpha } }} - 1$$

The elasticity for the BEW distribution is

10 $$\varepsilon \left( y \right) = \frac{{{ }\alpha {{\beta \lambda }}\left[ {1 - {\Upsilon }^{\beta } } \right]^{\alpha - 1} {\Upsilon }^{\beta - 1} }}{{1 - \left[ {1 - {\Upsilon }^{\beta } } \right]^{\alpha } }}$$

Mills ratio for the BEW distribution is

11 $$M\left( t \right) = \frac{{\left[ {1 - {\Upsilon }^{\beta } } \right]^{\alpha } y}}{{{\uplambda }\alpha {\upbeta }\left[ {1 - {\Upsilon }^{\beta } } \right]^{\alpha - 1} {\Upsilon }^{\beta } }}$$

By utilizing the mathematical expressions stated above, researchers can analyze or model the data using the survival, hazard, and reverse hazard characteristics associated with the BEW distribution.

**Figure 1:**
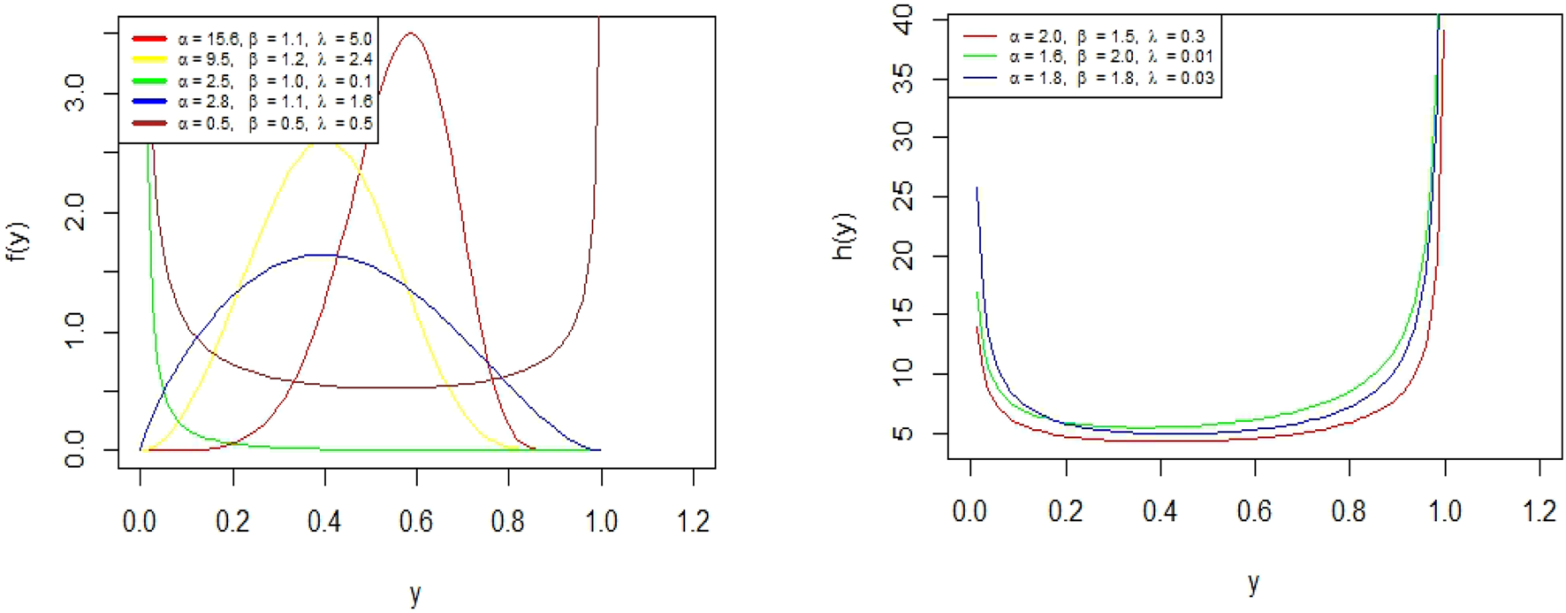
PDF (left) and HRF (right) of the BEWD.

From Fig. 1, the PDF graphs of BEW distribution show a variety of shapes, positively/negatively skewed, symmetrical, U shapes, reverse J shape. The hazard function of the BEW distribution shows bathtub shape.

### Some distributional properties of BEW distribution

In this section some fundamental distributional properties such as quantile function, median, inter quartile range, moments, moment generating function (mgf), mean, variance and standard deviation for the BEW distribution have been presented.

The quantile function (QF) is the inverse of the CDF of any PDF. The QF of the BEW distribution is as follow:

$$Q_{Y} \left( p \right) = F_{Y} \left( {y;\lambda ,\alpha ,\beta } \right) = q$$

12 $${\text{Q}}\left( {y;\lambda ,{ }\alpha ,\beta } \right) = y_{q} = \left[ {1{ } - { }p^{{{\raise0.7ex\hbox{$1$} \!\mathord{\left/ {\vphantom {1 \alpha }}\right.\kern-0pt} \!\lower0.7ex\hbox{$\alpha $}}}} } \right]^{{{\raise0.7ex\hbox{$1$} \!\mathord{\left/ {\vphantom {1 {\lambda \beta }}}\right.\kern-0pt} \!\lower0.7ex\hbox{${\lambda \beta }$}}}} ,{\text{ where}}, \;\;p = 1 - q.$$

The median and Inter quartile range (IQR) for the BEW distribution are calculated as Median = $y_{0.5}$ and IQR = $y_{0.75} - { }y_{0.25}$ in (12).

The $r{\text{th}}$ moments for the BEW distribution are defined as

13 $$\mu_{r}^{\prime } { } = { }\alpha {\text{B}}\left( {\frac{r}{\lambda \beta } + 1,{ }\alpha } \right)$$

The mean of the BEW distribution is

14 $$\mu_{1}{\prime} { } = { }\frac{{\alpha {\Gamma }\left( {1/\beta \lambda } \right){\Gamma }\left( \alpha \right)}}{{\left( {1{ } + { }\alpha \beta \lambda } \right){\Gamma }\left( {\frac{1}{\beta \lambda }{ } + { }\alpha } \right)}}$$

The variance of the BEW distribution is

15 $$\sigma^{2} = { }\frac{{2\alpha {\Gamma }\left( {2/\beta \lambda } \right){\Gamma }\left( \alpha \right)}}{{\left( {2{ } + { }\alpha \beta \lambda } \right){\Gamma }\left( {\frac{2}{\beta \lambda }{ } + { }\alpha } \right)}} - \left( {\frac{{\alpha {\Gamma }\left( {1/\beta \lambda } \right){\Gamma }\left( \alpha \right)}}{{\left( {1{ } + { }\alpha \beta \lambda } \right){\Gamma }\left( {\frac{1}{\beta \lambda }{ } + { }\alpha } \right)}}} \right)^{2}$$

The MGF for the BEW distribution is given below

16 $$M_{y} \left( t \right){ } = { }\alpha \mathop \sum \limits_{n = 0}^{\infty } \frac{{t^{n} }}{n!}{\text{ B}}\left( {\frac{r}{\beta \lambda } + 1,{ }\alpha } \right)$$

Numerical description of the standard deviation (SD), coefficient of Skewness (CV), and coefficient of Kurtosis (CK), coefficient of skewness (CS) for the BEW distribution for some parametric values are given in Table 1.

**Table 1:** Computed moments for different choices of $\alpha$, $\beta$, $\lambda$.

$\mu_{r}^{\prime }$	$\alpha$ = 20, $\beta$ = 5.3, $\lambda$ = 1.5	$\alpha$ = 0.4, $\beta$ = 0.9, $\lambda$ = 2.5	$\alpha$ = 4.5, $\beta$ = 2.5, $\lambda$ = 3.1
CK	0.000645	0.006940	0.000836
CS	0.005307	0.325116	0.013198
CV	1.421791	1.429206	1.421285
SD	0.094349	0.176989	0.107978
$\mu_{1}^{\prime }$	0.643616	0.857228	0.762535
$\mu_{2}^{\prime }$	0.423143	0.766165	0.593119
$\mu_{3}^{\prime }$	0.283305	0.701539	0.469095
$\mu_{4}^{\prime }$	0.192714	0.652533	0.376329

### Inequality measures

The Lorenz and Bonferroni curves are used in several fields of study, such as economics, demography, reliability, medicine, and insurance. They are commonly used to analyze the poverty and income values of imbalance. To find these inequalities we first need to derive incomplete moments.

#### Theorem 1.

An $r{\text{th}}$ incomplete central moment of BEW distribution is given below.

17 $$\varphi_{r} { } = { }\alpha {\text{B}}\left( {y^{{\uplambda }} ;{ }\frac{r}{\beta \lambda } + 1,{ }\alpha } \right)$$

where B $\left( {u;\alpha ,{ }\beta } \right)$ = $\mathop \smallint \limits_{0}^{u} y^{\alpha - 1} \left( {1 - y} \right)^{\beta - 1}$ dy, it is known as the incomplete beta function.

#### Proof.

Let the random variable Y follow the PDF given in Eq. (4), then the incomplete moments are.

$$\varphi_{r} = {\text{E}}\left( {y^{r} } \right) = \mathop \smallint \limits_{0}^{y} y^{r} f\left( y \right)\;\;{\text{dy}}$$

$$\varphi_{r} = \mathop \smallint \limits_{0}^{y} y^{r} { }\alpha {{\beta \lambda }}y^{ - 1} {{\Upsilon \Upsilon }}^{\beta - 1} \left[ {1 - {\Upsilon }^{\beta } } \right]^{\alpha - 1} \;\;{\text{dy}}$$

Let ${\text{z}} = {\Upsilon }^{\beta } ,$ and simplifying it we get.

$$\varphi_{r} = \alpha \mathop \smallint \limits_{0}^{y} z^{{\frac{r}{\beta \lambda } + 1 - 1}} \left( {1 - z} \right)^{\alpha - 1} \;\;{\text{dz}}$$

So, the above expression becomes the expression given in Eq. (17).

#### Theorem 2.

The Lorenz curve $L_{F} \left( y \right)$ for the BEW distribution is defined as

18 $${\text{L}}_{{\text{F}}} \left( {\text{y}} \right){ } = { }\frac{{\upalpha }}{{\upmu }}{\text{ B}}\left( {y;{ }\frac{1}{\beta \lambda } + 1,{ }\alpha } \right)$$

where ${\text{B}}\left( {z;\alpha ,{ }\beta } \right) = \mathop \smallint \limits_{0}^{z} y^{\alpha - 1} \left( {1 - y} \right)^{\beta - 1}$ dy, it is known as the incomplete beta function.

#### Proof.

Let $r = 1$ in the incomplete moments of BEW distribution derived in Theorem 1, and the Lorenz curve is defined as

$${\text{L}}_{{\text{F}}} \left( {\text{y}} \right) = \frac{1}{{\upmu }}{ }\mathop \smallint \limits_{0}^{{\text{y}}} {\text{y f}}\left( {\text{y}} \right)dy$$

By simplifying it we get the expression given in (18).

#### Theorem 3.

Bonferroni curve ${\text{B}}_{F} \left( y \right)$ for the BEW distribution is defined as

19 $${\text{B}}_{{\text{F}}} \left( {\text{y}} \right) = \frac{{\frac{{\upalpha }}{{\upmu }}{\text{ B}}\left( {y;{ }\frac{1}{\beta \lambda } + 1,{ }\alpha } \right)}}{{1{ } - { }\left[ {1 - {\Upsilon }^{\beta } } \right]^{\alpha } }}$$

#### Proof:

let Lorenz curve derived in Theorem 2, and the CDF of the BEW distribution given in Eq. (3), in the following expression

$${\text{B}}_{{\text{F}}} \left( {\text{y}} \right) = \frac{{{\text{L}}_{{\text{F}}} \left( {\text{y}} \right)}}{{{\text{F}}\left( {\text{y}} \right)}}$$

By simplifying this we get the results given in Eq. (19).

## Generalization of proposed methodology: a bivariate version of a BEW distribution

Many researchers are making prognostications regarding the relationship between the two numerical variables in a dataset, such as the correlation between an individual's age and BMI. Bivariate distributions serve as a valuable tool to observe the independence between variables and evaluate the dependability of products, particularly in insurance risk analysis, economics, and waiting time analysis. Within this section, an extended form of the BEW distribution known as the bivariate bounded exponentiated Weibull distribution (B-BEWD), is presented. We provide a illustration of CDF and PDF of the B-BEWD below.

20 $$F_{X,Y} \left( {x,y;{\varvec{\eta}}{ }} \right){ } = \frac{{\left[ {1{ } - { }\left( {1 - \left( {x^{{\uplambda }} } \right)^{\beta } } \right)^{\alpha } } \right]\left[ {1{ } - { }\left( {1 - {\Upsilon }^{\beta } } \right)^{\alpha } } \right]}}{{\left\{ {1{ } - { }\left( {\delta_{1} + \delta_{3} } \right)\left( {1 - \left( {x^{{\uplambda }} } \right)^{\beta } } \right)^{\alpha } + { }\left( {\delta_{2} + \delta_{3} } \right)\left( {1 - {\Upsilon }^{\beta } } \right)^{\alpha } } \right\}^{ - 1} }}$$

where $\lambda ,{ }\alpha ,\beta > 0$, ${ } - 1 < \delta_{{3{ }}} + \delta_{1} < 1$, $- 1 < \delta_{3} + \delta_{2} < 1$, $0 < {\text{y}} < 1$, $0 < {\text{ x}} < 1{ }$ and ${\varvec{\eta}}{ } = { }\left( {\lambda ,\delta_{1} ,\delta_{2} ,\delta_{3} ,\alpha ,\beta ,{ }} \right)^{{\varvec{T}}}$. The constraints $\delta_{1} ,\delta_{2,}$ and $\delta_{3}$ estimate the dependency or independency between a B-BWD random variables.

21 $$f_{X,Y} \left( {x,y;{\varvec{\eta}}{ }} \right){ } = { }\frac{{\left( {\alpha \beta {\uplambda }} \right)^{2} \left( {xy} \right)^{{{{\lambda \beta }}}} \left[ {\left( {1 - \left( {x^{{\uplambda }} } \right)^{\beta } } \right)\left( {1 - {\Upsilon }^{\beta } } \right)} \right]^{\alpha } \left[ {\left( {2\delta_{2} + 2\delta_{3} } \right)\left( {1 - {\Upsilon }^{\beta } } \right)^{\alpha } - { }\left( {2\delta_{1} + 2\delta_{3} } \right)\left( {1 - \left( {x^{{\uplambda }} } \right)^{\beta } } \right)^{\alpha } - { }\delta_{2} + \delta_{1} + 1} \right]}}{{xy\left( {1 - \left( {x^{{\uplambda }} } \right)^{\beta } } \right)\left( {1 - {\Upsilon }^{\beta } } \right)}}$$

**Figure 2:**
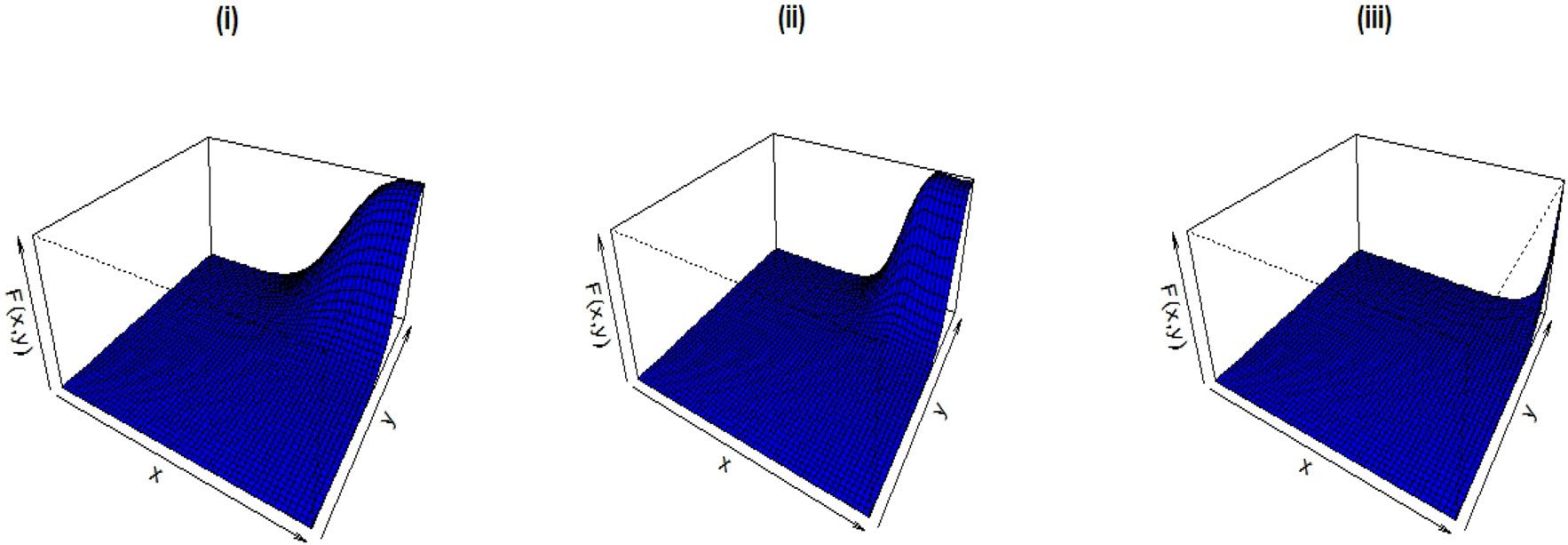
CDF plots of the B-BEW distribution.

Figure 2 shows the CDF graphs for the given parameter values.

(i) λ = 6.0, $$\alpha$$ = 4.5, $$\beta$$ = 1.3, $$\delta_{1}$$ = 0.5, $$\delta_{2}$$ = 0.2, $$\delta_{3}$$ = 0.4;
(ii) λ = 10.5, $$\alpha$$ = 9.5, $$\beta$$ = 1.3, $$\delta_{1}$$ = 0.2, $$\delta_{2}$$ = 0.9, $$\delta_{3}$$ = 0.4 and
(iii) λ = 4.8, $$\alpha$$ = 0.5, $$\beta$$ = 2.1, $$\delta_{1}$$ = − 0.3, $$\delta_{2}$$ = − 0.7, $$\delta_{3}$$ = − 0.1.

**Figure 3:**
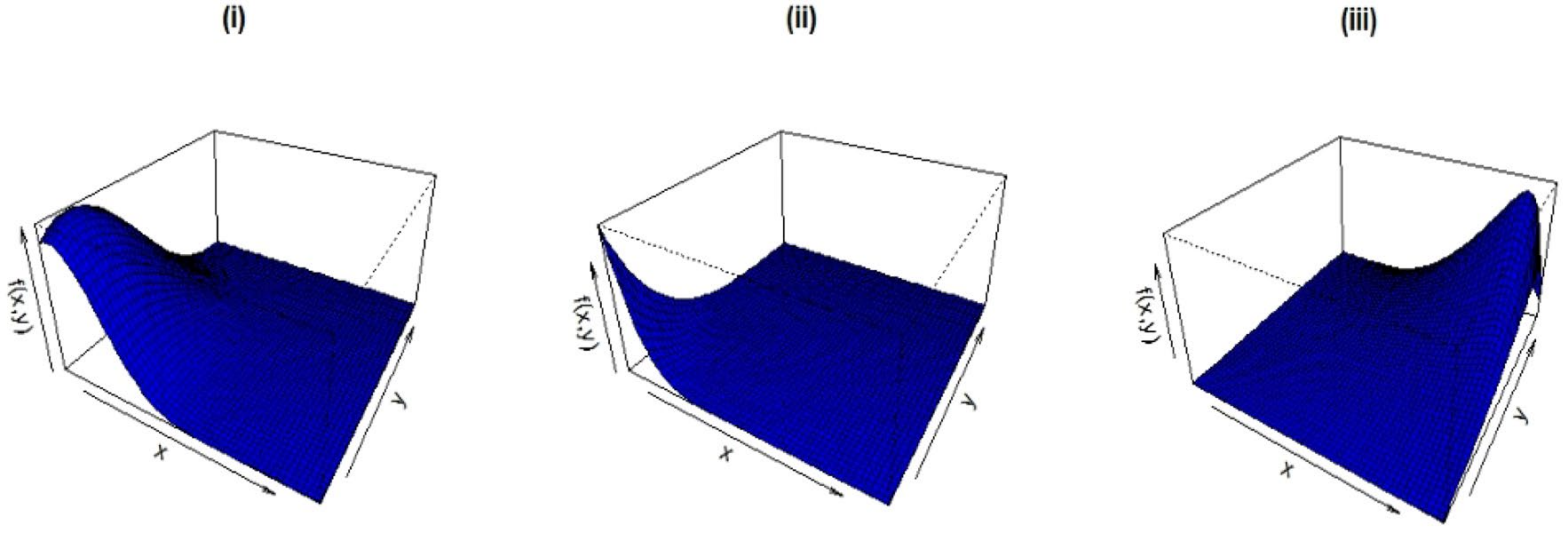
PDF plots of the B-BEW distribution.

Figure 3 shows the PDF plots for the given parameter values.

(i) λ = 1.0, $$\delta_{1}$$ = 0.5, $$\delta_{2}$$ = 0.2, $$\delta_{3}$$ = 0.4, $$\alpha$$ = 2.5, $$\beta$$ = 1.3;
(ii) λ = 0.5, $$\delta_{1}$$ = 0.2, $$\delta_{2}$$ = 0.9, $$\delta_{3}$$ = 0.4, $$\alpha$$ = 2.5, $$\beta$$ = 1.3 and
(iii) λ = 5.8, $$\delta_{1}$$ = − 0.3, $$\delta_{2}$$ = − 0.7, $$\delta_{3}$$ = − 0.1, $$\alpha$$ = 4.5, $$\beta$$ = 4.1,

## Parameter estimation methods

Six different methods for the estimation of the parameters have been covered in this section. These methods include maximum likelihood estimation (MLE) Cramér-von Mises estimation (CVME), ordinary least squares estimation (OLSE), weighted least squares estimation (WLSE), Percentile estimation (PC), and Anderson-Darling estimation (ADE) methods.

### Maximum Likelihood Estimation

In this section the parameters of the BEW distribution are estimated by the MLE. Let $Y_{1} ,Y_{2} , \ldots , Y_{n}$ be a random sample of size *n* and let $y_{1} ,y_{2} , \ldots , y_{n}$ be a random sample values from the BEW distribution the likelihood function (L) is:

we have

$${\text{L}}\left( \vartheta \right) = {\text{L}}\left( {y_{1} ,y_{2} , \ldots , y_{n} ;\alpha ,\beta ,\lambda } \right){ } = \left( {a\beta \lambda } \right)^{n} \mathop \sum \limits_{i = 1}^{n} {\Upsilon }y^{ - 1} \mathop \sum \limits_{i = 1}^{n} {\Upsilon }^{\beta - 1} \mathop \sum \limits_{i = 1}^{n} \left( {1 - {\Upsilon }} \right)^{\alpha - 1}$$

Then applying the log-likelihood function $l = l\left( \vartheta \right),{\text{ where }}\vartheta = \alpha ,\beta , {\text{and}} \lambda .$

22 $$\begin{aligned} l\left( \vartheta \right) & = l\left( {y_{1} ,y_{2} , \ldots , y_{n} ;\alpha ,\beta ,\lambda } \right){ } = { }nlog\left( {\alpha \beta {{ \lambda }}} \right) + \mathop \sum \limits_{i = 1}^{n} \left( {\lambda - 1} \right)\log \left( y \right) \\ & \;\;\;\; + \mathop \sum \limits_{i = 1}^{n} \left( {\beta - 1} \right)\log \left( {\Upsilon } \right) + \mathop \sum \limits_{i = 1}^{n} \left( {\alpha - 1} \right)log\left( {1 - {\Upsilon }} \right) \\ \end{aligned}$$

To estimate the values of the parameters of BEW, taking derivative of Eq. (22) with respect to $\alpha ,\beta { }and{ }\lambda$ respectively, and we obtain

$$\frac{d\ell }{{d\alpha }}{ } = { }\frac{n}{\alpha } + \mathop \sum \limits_{i = 1}^{n} log\left( {1 - {\Upsilon }} \right)$$

$$\frac{d\ell }{{d\beta }} = \frac{n}{\alpha } + \mathop \sum \limits_{i = 1}^{n} log\left( {\Upsilon } \right)$$

$$\frac{d\ell }{{d\lambda }}{ } = \frac{{\text{n}}}{\lambda } + \mathop \sum \limits_{i = 1}^{n} log\left( y \right) + \left( {\beta - 1} \right)\mathop \sum \limits_{i = 1}^{n} \left( {\frac{\lambda }{y}} \right) - \left( {\alpha - 1} \right)\mathop \sum \limits_{i = 1}^{n} \frac{{\lambda {\Upsilon }y^{ - 1} }}{{\left( {1 - {\Upsilon }} \right)}}$$

Because the above equations do not have a closed form, the non-linear system of equations $\left( {\frac{{d\ell_{n}^{*} }}{d\alpha },\frac{{d\ell_{n}^{*} }}{d\lambda }} \right){ }^{T} = \left( {0,0} \right)^{T}$ therefore these equations can be numerically solved to find the parameter estimates.

### Ordinary and Weighted Least Squares Estimation Methods

Let $Y_{1} ,Y_{2} , \ldots ,Y_{n}$ be the ordered values from the BEW distribution with distribution function F(Y). For a sample of size n, we have ${\text{E}}\left[ {F\left( {Y_{\left( i \right)} } \right)} \right]{ } = { }\frac{i}{{\left( {n + 1} \right)}}$. The least-square estimator parameters $\alpha$, $\beta$ and $\lambda$ for the BEW distribution are estimated by minimizing.

23 $$Q\left( {\alpha ,\beta ,\lambda } \right){ } = \mathop \sum \limits_{i = 1}^{n} \left[ {F\left( {Y_{{\left( {i:n} \right)}} |\alpha ,\lambda ,\beta } \right) - { }\frac{i}{{\left( {n + 1} \right)}}} \right]^{2}$$

In the case of BEW distribution, Eq. (23) becomes.

24 $$Q\left( {\alpha ,\beta ,\lambda } \right){ } = \mathop \sum \limits_{i = 1}^{n} \left[ {1{ } - { }\left\{ {1 - \left( {y_{\left( i \right)}^{\lambda } } \right)^{\beta } } \right\}^{\alpha } - { }\frac{i}{{\left( {n + 1} \right)}}} \right]^{2}$$

Take the partial derivative of (24) with respect to the parameters to determine the estimates for $\alpha ,$ $\beta ,$ and $\lambda .$ The following equations are,

25 $$\mathop \sum \limits_{i = 1}^{n} \left[ {1{ } - { }\left\{ {1 - \left( {y_{\left( i \right)}^{\lambda } } \right)^{\beta } } \right\}^{\alpha } - { }\frac{i}{{\left( {n + 1} \right)}}} \right]\Delta_{s} \left( {y_{\left( i \right)} |\alpha ,\beta ,\lambda } \right) = { }0,{\text{ s }} = { }1,{ }2,{ }3$$

where

$$\Delta_{1} \left( {y_{\left( i \right)} |\alpha } \right) = - 2{\text{ln}}\left( {1 - \left( {y_{\left( i \right)}^{\lambda } } \right)^{\beta } } \right)\left( {1 - \left( {y_{\left( i \right)}^{\lambda } } \right)^{\beta } } \right)^{\alpha } ,$$

$$\Delta_{2} \left( {y_{\left( i \right)} |\beta } \right){ } = { }2\alpha {\text{ln}}\left( {y_{\left( i \right)}^{\lambda } } \right)\left( {y_{\left( i \right)}^{\lambda } } \right)^{\beta } \left( {1 - \left( {y_{\left( i \right)}^{\lambda } } \right)^{\beta } } \right)^{\alpha - 1}$$

and

$$\Delta_{3} \left( {y_{\left( i \right)} |\lambda } \right){ } = { }2\alpha \beta {\text{ln}}\left( {y_{\left( i \right)} } \right)\left( {y_{\left( i \right)}^{\lambda } } \right)^{\beta } \left( {1 - \left( {y_{\left( i \right)}^{\lambda } } \right)^{\beta } } \right)^{\alpha - 1} .$$

By simplifying Eq. (25) the WLS estimates $\hat{\alpha }_{WLS}$, $\hat{\beta }_{WLS}$ and $\hat{\lambda }_{WLS}$, can obtain by minimizing.

26 $${\text{WLS}}\left( {\alpha ,\beta ,\lambda } \right){ } = \mathop \sum \limits_{i = 1}^{n} \frac{{\left( {n + 1} \right)^{2} \left( {n + 2} \right)}}{{i\left( {n - i + 1} \right)}}\left[ {F\left( {Y_{{\left( {i:n} \right)}} |\alpha ,\beta ,\lambda } \right) - { }\frac{i}{{\left( {n + 1} \right)}}} \right]^{2}$$

27 $${\text{WLS}}\left( {\alpha ,\beta ,\lambda } \right){ } = { }\mathop \sum \limits_{i = 1}^{n} \frac{{\left( {n + 1} \right)^{2} \left( {n + 2} \right)}}{{i\left( {n - i + 1} \right)}}\left[ {1{ } - { }\left\{ {1 - \left( {y_{\left( i \right)}^{\lambda } } \right)^{\beta } } \right\}^{\alpha } - { }\frac{i}{{\left( {n + 1} \right)}}} \right]^{2}$$

Take the partial derivative of Eq. (27) with respect to the parameters $\hat{\user2{\alpha }}$,$\user2{ \hat{\beta }}$ and $\hat{\user2{\lambda }}$. The following equations are:

28 $$\mathop \sum \limits_{i = 1}^{n} \frac{{\left( {n + 1} \right)^{2} \left( {n + 2} \right)}}{{i\left( {n - i + 1} \right)}}\left[ {1{ } - { }\left\{ {1 - \left( {y_{\left( i \right)}^{\lambda } } \right)^{\beta } } \right\}^{\alpha } - \frac{i}{{\left( {n + 1} \right)}}} \right]\Delta_{s} \left( {y_{\left( i \right)} |\alpha ,\lambda } \right) = { }0,{\text{ s }} = { }1,{ }2,{ }3$$

where, $\Delta_{s} \left( {y_{\left( i \right)} |\alpha ,{\upbeta },\lambda } \right) = { }0,{\text{ s }} = { }1,{ }2,{ }3$ is defined above.

### Cramér–Von Mises estimation

Let $Y_{1} ,Y_{2} , \ldots ,Y_{n}$ be the ordered values arise from the BEW distribution. The Cramér–Von Mises is used to find the parameters $\hat{\alpha }_{CVM}$, $\hat{\beta }_{CVM}$, and $\hat{\lambda }_{CVM}$ that are find out by minimizing the function that is given below.

29 $${\text{CVM}}\left( {\alpha ,\beta ,\lambda } \right){ } = { }\frac{1}{12n}{ } + \mathop \sum \limits_{i = 1}^{n} \left[ {F\left( {Y_{{\left( {i:n} \right)}} |\alpha ,\lambda } \right) - { }\frac{2i - 1}{{2n}}} \right]^{2}$$

30 $${\text{CVM}}\left( {\alpha ,\beta ,\lambda } \right){ } = { }\frac{1}{12n}{ } + \mathop \sum \limits_{i = 1}^{n} \left[ {1{ } - { }\left\{ {1 - \left( {y_{\left( i \right)}^{\lambda } } \right)^{\beta } } \right\}^{\alpha } - { }\frac{2i - 1}{{2n}}} \right]^{2} .$$

Differentiate the Eq. (30) with respect to $\alpha$, $\beta$ and $\lambda$, the estimates of the parameters can be determined numerically by the equations given below.

31 $$\mathop \sum \limits_{i = 1}^{n} \left[ {1{ } - { }\left\{ {1 - \left( {y_{\left( i \right)}^{\lambda } } \right)^{\beta } } \right\}^{\alpha } - { }\frac{2i - 1}{{2n}}} \right]\Delta_{s} \left( {y_{\left( i \right)} |\alpha ,\lambda } \right) = { }0,{\text{ s }} = { }1,{ }2,{ }3,$$

where, $\Delta_{s} \left( {y_{\left( i \right)} |\alpha ,\beta ,\lambda } \right)$ are defined in the section “Ordinary and Weighted Least Squares estimation methods”.

### Anderson–Darling estimation

Let $Y_{1} ,Y_{2} , \ldots ,Y_{n}$ be ordered observations arise from BEW distribution. The Anderson–Darling is determined by minimizing the function that are given below to find the parameters $\hat{\alpha }_{AD}$ and $\hat{\lambda }_{AD}$.

32 $${\text{A}}\left( {\alpha ,\beta ,\lambda } \right){ } = { } - n - \frac{1}{n}{ }\mathop \sum \limits_{i = 1}^{n} \left( {2i - 1} \right)\left\{ {logF\left( {x_{1:n} |\alpha ,\lambda } \right) + log\overline{F}\left( {x_{n + 1 - i:n} |\alpha ,\lambda } \right)} \right\}.$$

These estimators can be derived by solving the non-linear equations that are given below.

33 $${\text{A}}\left( {\alpha ,\beta ,\lambda } \right){ } = { } - n - \frac{1}{n}{ }\mathop \sum \limits_{i = 1}^{n} \left( {2i - 1} \right)\left[ {log\left\{ {1{ } - { }\left( {1 - \left( {y_{\left( i \right)}^{\lambda } } \right)^{\beta } } \right)} \right\} + log\left( {1 - \left( {y_{\left( i \right)}^{\lambda } } \right)^{\beta } } \right)} \right].$$

### Percentile estimation

Let $Y_{1} ,Y_{2} , \ldots ,Y_{n}$ be ordered observations came from BEW distribution and $u_{i}$ = $\frac{i}{n + 1}$ is an unbiased estimate of $F_{Y} \left( {y_{\left( i \right)} ;\alpha ,\beta ,\lambda } \right)$. The PC estimates for the BEW distribution parameters are derived by minimizing the following function:

34 $${\text{PC}}\left( {\alpha ,\beta ,\lambda } \right){ } = \mathop \sum \limits_{i = 1}^{n} \left[ {y_{i} - {\text{ Q}}\left( {y;\alpha ,\lambda } \right)} \right]^{2} ,$$

35 $${\text{PC}}\left( {\alpha ,\beta ,\lambda } \right){ } = \mathop \sum \limits_{i = 1}^{n} \left[ {y_{i} - { }\left[ {1{ } - { }\left( {1 - q} \right)^{{{\raise0.7ex\hbox{$1$} \!\mathord{\left/ {\vphantom {1 \alpha }}\right.\kern-0pt} \!\lower0.7ex\hbox{$\alpha $}}}} } \right]^{{{\raise0.7ex\hbox{$1$} \!\mathord{\left/ {\vphantom {1 {\lambda \beta }}}\right.\kern-0pt} \!\lower0.7ex\hbox{${\lambda \beta }$}}}} } \right]^{2} .$$

## Simulation study

In this section a simulation study is represented by using the BEW distribution to assess the performance of the estimators discussed in the previous section and numerical results are obtained. We generate N = 10,000 samples of the size n = (20, 40, 100, 300) from BEW distribution with parameter settings $\left( {\alpha = 1,{ }\beta = 2,{ }\lambda = 3{\text{ and }}\alpha = 1.7,{ }\beta = 0.5,{ }\lambda = 2.8} \right)$. The random numbers generation is obtained by quantile function of BEW distribution. In this simulation study, we calculate the empirical mean, bias, and mean square errors (MSE’s) of all estimators to compare in the terms of their biases and MSE’s with varying sample size.

$$\widehat{{Bias_{i} }} = \mathop \sum \limits_{i = 1}^{N} \left( {\widehat{{t_{i} }} - t} \right)$$

and

$$\widehat{{MSE_{t} }} = \frac{1}{N}\mathop \sum \limits_{i = 1}^{N} \left( {\widehat{{t_{i} }} - t} \right)^{2} .$$

In Tables 2 and 3 the simulations study with the help of bias, average bias, MSE and mean relative error (MRE) are shown for small, medium, and large ample sizes. The proposed estimation methods are used such as maximum likelihood estimator (MLE), Anderson Darling (AD), Cramer-von Mises (CVM), ordinary least square (OLS) and weighted least square (WLS). It is observed that for large (n = 300) and for medium (*n* = 100) sample sizes MLE is performing better as compared to AD, CVM, OLS and WLS. For small (*n* = 20) and (*n* = 50) AD is better than MLE, CVM, OLS and WLS and MLE is better than CVM, OLS and WLS.

**Table 2:** The Bias, Average Bias, MSE, and MRE for ${\varvec{\alpha}} = 1$, ${\varvec{\beta}} = 2$, ${\varvec{\lambda}} = 3$.

Methods		$\alpha = 1$				$\beta = 2$				$\lambda = 3\lambda = 3$			
		20	50	100	300	20	50	100	300	20	50	100	300
MLE	Average Bias	1.8182	1.6306	1.5624	1.5308	1.5091	1.0489	1.0229	1.0094	1.0051	1.0026	1.0489	1.0229
	Bias	0.5040	0.3032	0.1803	0.1267	0.0852	0.1145	0.0760	0.0488	0.0339	0.0243	0.1145	0.0760
	MSE	0.6838	0.1758	0.0547	0.0265	0.0115	0.0226	0.0096	0.0038	0.0018	0.0009	0.0226	0.0096
	MRE	0.3360	0.2022	0.1202	0.0845	0.0568	0.1145	0.0760	0.0488	0.0339	0.0243	0.1145	0.0760
ADE	Average Bias	0.2485	0.1138	0.0451	0.0237	0.0111	0.0181	0.0098	0.0039	0.0019	0.0008	0.0181	0.0098
	Bias	0.4549	0.3040	0.1850	0.1267	0.0942	0.1138	0.0779	0.0507	0.0369	0.0253	0.1138	0.0779
	MSE	0.4628	0.1711	0.0585	0.0262	0.0139	0.0206	0.0101	0.0041	0.0021	0.0010	0.0206	0.0101
	MRE	0.3033	0.2027	0.1233	0.0844	0.0628	0.1138	0.0779	0.0507	0.0369	0.0253	0.1138	0.0779
CVME	Average Bias	1.9079	1.6638	1.5745	1.5404	1.5148	1.0542	1.0249	1.0093	1.0063	1.0035	1.0542	1.0249
	Bias	0.6442	0.3825	0.2262	0.1533	0.1027	0.1351	0.0929	0.0598	0.0399	0.0283	0.1351	0.0929
	MSE	1.4938	0.3208	0.0897	0.0394	0.0171	0.0326	0.0147	0.0056	0.0025	0.0013	0.0326	0.0147
	MRE	0.4295	0.2550	0.1508	0.1022	0.0685	0.1351	0.0929	0.0598	0.0399	0.0283	0.1351	0.0929
OLSE	Average Bias	1.6593	1.5673	1.5002	1.5134	1.5039	1.0052	1.0017	0.9952	0.9986	1.0005	1.0052	1.0017
	Bias	0.5332	0.3490	0.2000	0.1453	0.1078	0.1207	0.0913	0.0570	0.0392	0.0288	0.1207	0.0913
	MSE	1.2373	0.2686	0.0668	0.0343	0.0181	0.0242	0.0135	0.0051	0.0025	0.0013	0.0242	0.0135
	MRE	0.3555	0.2326	0.1334	0.0969	0.0719	0.1207	0.0913	0.0570	0.0392	0.0288	0.1207	0.0913
WLSE	Average Bias	1.7128	1.5358	1.5115	1.5149	1.5087	1.0052	0.9990	0.9988	1.0041	0.9999	1.0082	0.9993
	Bias	0.5473	0.3077	0.1935	0.1309	0.0946	0.1186	0.0828	0.0508	0.0380	0.0253	0.1209	0.0830
	MSE	4.6260	0.1699	0.0616	0.0271	0.0143	0.0322	0.0111	0.0040	0.0023	0.0010	0.0840	0.0112
	MRE	0.3649	0.2051	0.1290	0.0873	0.0631	0.1186	0.0828	0.0508	0.0380	0.0253	0.1209	0.0830

**Table 3:** The Bias, Average Bias, MSE, and MRE for ${\varvec{\alpha}} = 1.7$, ${\varvec{\beta}} = 0.5$, ${\varvec{\lambda}} = 2.8$.

Methods		$\alpha = 1.7$				$\beta = 0.5$				$\lambda = 2.8\lambda = 2.8$			
		20	50	100	300	20	50	100	300	20	50	100	300
MLE	Average Bias	0.9934	0.9994	1.0000	1.0000	0.9562	0.9737	0.9835	0.9938	0.9926	0.9952	0.9953	0.9961
	Bias	0.7066	0.7006	0.7000	0.7000	0.4562	0.4737	0.4835	0.4938	1.8074	1.8048	1.8047	1.8039
	MSE	0.5001	0.4908	0.4900	0.4900	0.2132	0.2266	0.2348	0.2440	3.2671	3.2575	3.2569	3.2540
	MRE	0.4156	0.4121	0.4118	0.4118	0.9124	0.9474	0.9669	0.9876	0.6455	0.6446	0.6445	0.6442
ADE	Average Bias	0.9779	0.9950	0.9989	1.0000	0.9004	0.9191	0.9259	0.9268	0.9881	0.9890	0.9915	0.9958
	Bias	0.7221	0.7050	0.7011	0.7000	0.4004	0.4191	0.4259	0.4268	1.8119	1.8110	1.8085	1.8042
	MSE	0.5244	0.4975	0.4915	0.4900	0.1725	0.1817	0.1847	0.1839	3.2832	3.2799	3.2709	3.2552
	MRE	0.4248	0.4147	0.4124	0.4118	0.8009	0.8382	0.8518	0.8536	0.6471	0.6468	0.6459	0.6444
CVME	Average Bias	2.1893	1.8531	1.7648	1.7255	0.5835	0.5291	0.5118	0.5051	2.7450	2.7816	2.7895	2.7953
	Bias	0.8090	0.3879	0.2515	0.1434	0.1623	0.0863	0.0559	0.0323	0.0651	0.0272	0.0148	0.0076
	MSE	3.3679	0.3044	0.1118	0.0335	0.0948	0.0140	0.0053	0.0017	0.0255	0.0039	0.0009	0.0003
	MRE	0.4759	0.2282	0.1480	0.0843	0.3245	0.1726	0.1118	0.0645	0.0232	0.0097	0.0053	0.0027
OLSE	Average Bias	1.8448	1.7383	1.7147	1.7063	0.5160	0.5039	0.5013	0.5011	2.7536	2.7823	2.7891	2.7953
	Bias	0.6441	0.3543	0.2445	0.1407	0.1401	0.0806	0.0550	0.0324	0.0537	0.0252	0.0149	0.0077
	MSE	1.4935	0.2274	0.1017	0.0318	0.0576	0.0111	0.0050	0.0017	0.0174	0.0032	0.0009	0.0003
	MRE	0.3789	0.2084	0.1438	0.0828	0.2802	0.1612	0.1100	0.0649	0.0192	0.0090	0.0053	0.0028
WLSE	Average Bias	1.8502	1.7543	1.7297	1.7105	0.5222	0.5027	0.5022	0.5007	2.7868	2.8200	2.8107	2.8044
	Bias	0.5891	0.3261	0.2185	0.1243	0.1332	0.0687	0.0467	0.0271	0.1626	0.0965	0.0654	0.0467
	MSE	0.9526	0.2000	0.0822	0.0247	0.0389	0.0082	0.0036	0.0012	0.1525	0.0297	0.0126	0.0065
	MRE	0.3465	0.1918	0.1285	0.0731	0.2664	0.1374	0.0935	0.0542	0.0581	0.0345	0.0234	0.0167

For the numerical solutions, simulations of the estimation methods including MLE, AD, CVM, OLS, WLS, further analysis and applications, R studio^17^, and Wolfram MATHEMATICA 13.3 software are used.

## Applications

This section determines the significance of the BEW distribution and compares its performance with the other competing unit interval distributions. The databases belong to the unit interval observations as the mortality rate of COVID-19 patient (1) in Canada and (2) in the UK, and recovery rate (3) in Spain.

**Figure 4:**
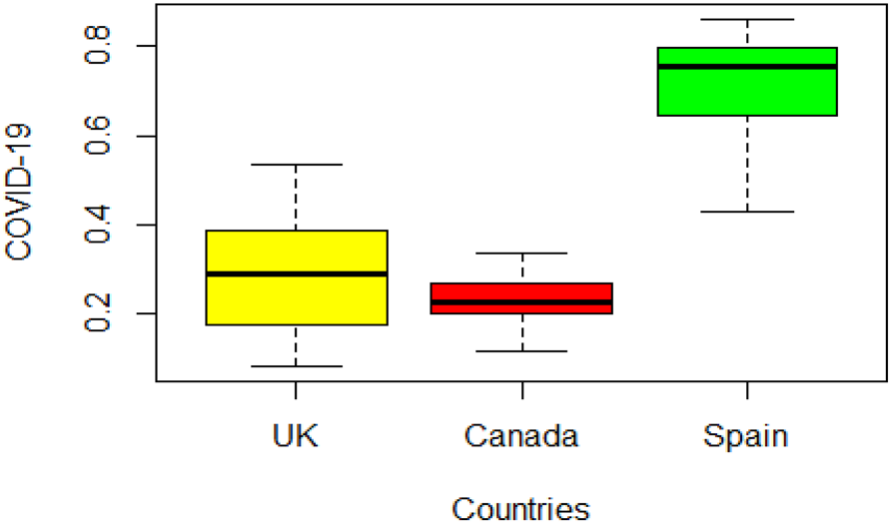
Box Plot of Covid-19 datasets.

Table 4 explains some descriptive measures for the mortality and recovery rate of COVID-19 data in three countries. The UK data is positive skewed, and Canada and Spain are negatively skewed similar trend is shown by the box plot in Fig. 4.

**Table 4:** Descriptive information for the mortality rate and recovery rate for COVID-19 patients in the stated countries.

Countries	UK	Canada	Spain
Minimum	0.0807	0.1159	0.4286
Maximum	0.5331	0.3347	0.8628
Mean	0.2888	0.2305	0.7240
Skewness	0.0489	− 0.0873	− 0.7049
Kurtosis	1.9616	2.6537	2.6021

**Figure 5:**
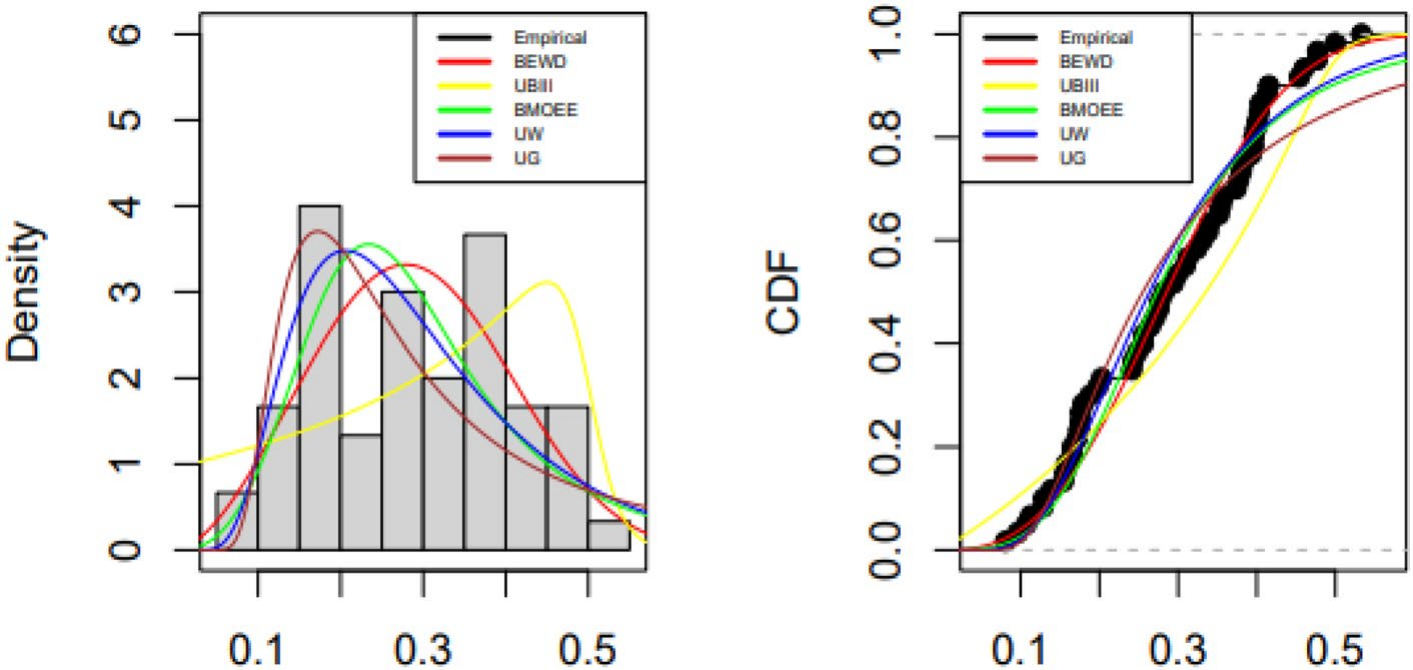
Fitted and empirical PDFs and CDFs of the UK dataset.

**Figure 6:**
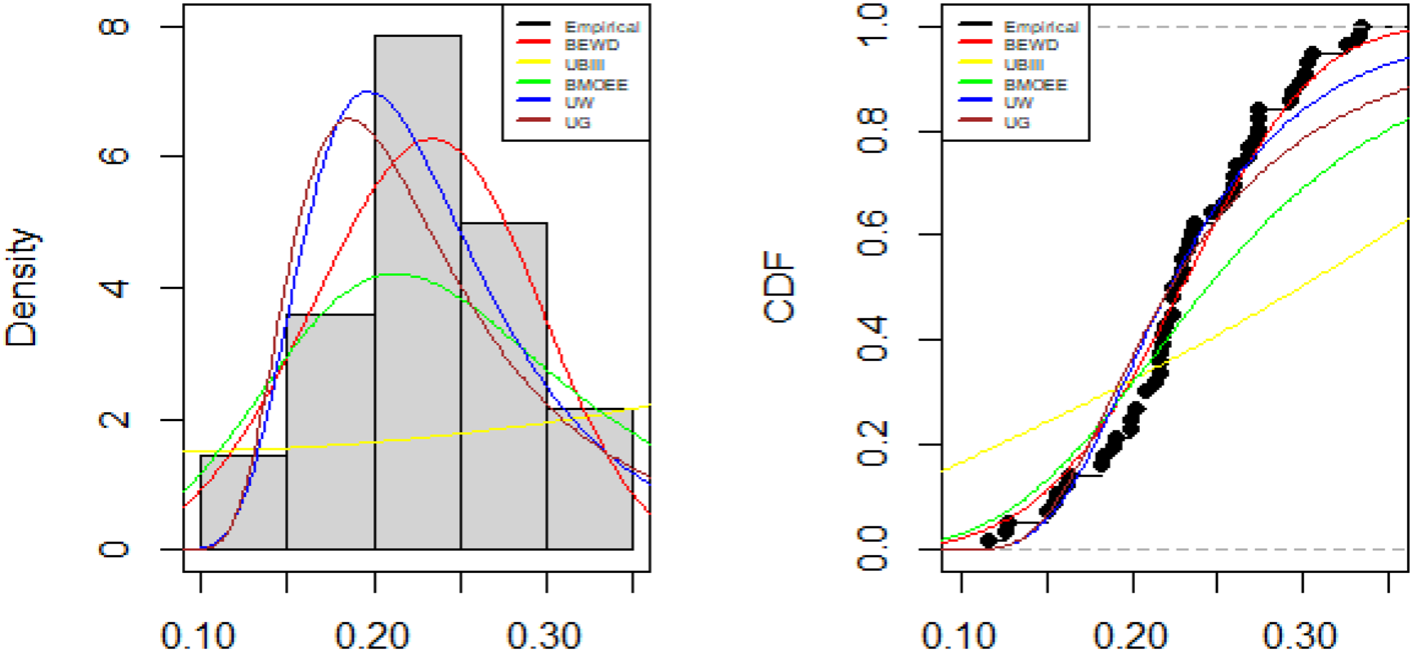
Empirical and fitted PDFs and CDFs of Canada dataset.

**Figure 7:**
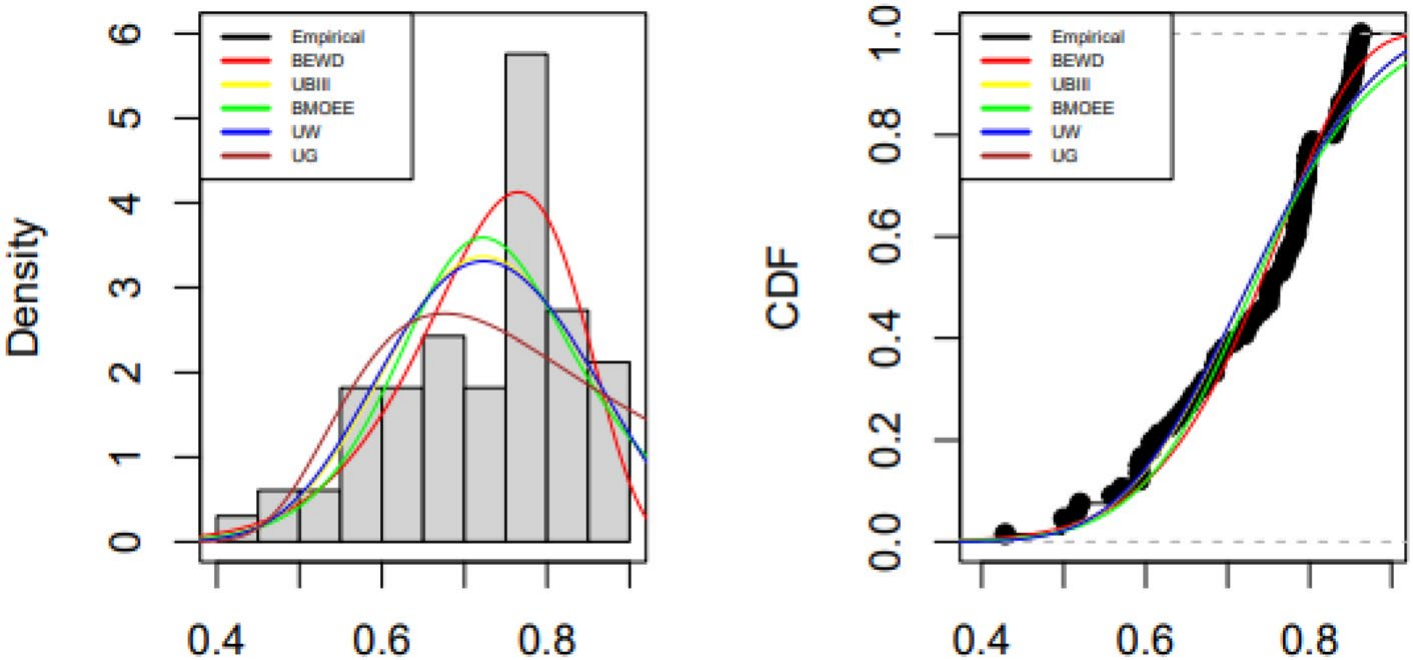
Fitted and empirical PDFs and CDFs of Spain dataset.

Tables 5, 6 and 7 contain the fitted distributions' along with values of test statistics with p-values and also the estimated values of the parameters by MLE of the parameters along with their standard errors. Figures 5, 6 and 7 show the comparison of empirical and fitted PDFs and CDFs for the three data sets.

**Table 5:** Model Selection Criteria and Parameter Estimates for UK.

Model	Parameters	$\ell$	AIC	BIC	CAID	AD	KS
BEWD	$\alpha =$ 19.5966 (6.5056) $\beta =$ 1.6374 (50.7632) $\lambda =$ 1.6374 (50.7632)	− 45.8644	− 85.7288	− 79.4458	0.0984	0.5955	0.1055 (0.4843)
UBIII	$\alpha = { }$ 0.0758 (0.0382) $\beta = { }$ 13.3691 (6.5426)	− 38.9028	− 73.8056	− 69.6170	0.0589	0.3377	0.1932 (0.0195)
BMOEE	$\alpha =$ 3.5711 (0.4035) $\beta =$ 101.7405 (57.0214)	− 40.7201	− 77.4402	− 73.2515	0.0916	0.6014	0.1034 (0.5092)
UW	$\alpha =$ 3.1229 (0.3047) $\beta =$ 0.2834 (0.0602)	− 42.5622	− 81.1244	− 76.9357	0.2312	1.4315	0.0955 (0.6105)
UG	$\alpha =$ 1.8208 (0.2198) $\beta =$ 0.0630 (0.0274)	− 36.4368	− 68.8736	− 64.6849	0.3677	2.2076	0.1309 (0.2338)

**Table 6:** Model selection criteria and parameter estimates for Canada.

Model	Parameters	$\ell$	AIC	BIC	CAID	AD	KS
BEWD	$\alpha =$ 302.8525 (129.2633) $\beta = { }$ 1.8551 $\lambda =$ 2.2226	− 84.8625	− 163.7250	− 157.6490	0.0601	0.3336	0.1077 (0.5341)
UBIII	$\alpha = { }$ 0.0721 (0.1606) $\beta = { }$ 11.2663 (25.0812)	− 30.8857	− 57.7715	− 53.7208	0.0589	0.3377	0.4336 (0.0000)
BMOEE	$\alpha =$ 3.8284 (0.2514) $\beta =$ 219.6406 (73.8469)	− 69.6576	− 135.3151	− 131.2644	0.0916	0.6014	0.2437 (0.0026)
UW	$\alpha =$ 6.1130 (0.5832) $\beta =$ 0.0567 (0.0197)	− 79.9508	− 155.9015	− 151.8508	0.2312	1.4315	0.1433 (0.2005)
UG	$\alpha =$ 3.4261 (0.1839) $\beta =$ 0.0040 (0.0013)	− 74.3143	− 144.6285	− 140.5778	0.3677	2.2076	0.1543 (0.1388)

**Table 7:** Model selection criteria and parameter estimates for Spain.

Model	Parameters	$\ell$	AIC	BIC	CAID	AD	KS
BEWD	$\alpha =$ 7.7384 (2.0186) $\beta = { }$ 2.8422 (—) $\lambda =$ 2.8422 (—)	− 58.8343	− 111.6686	− 105.0997	− 109.0729	− 111.2815	0.1366 (0.5278)
UBIII	$\alpha = { }$ 0.0721 (0.1606) $\beta = { }$ 11.2663 (25.0812)	− 53.7963	− 103.5927	− 99.2134	− 101.8622	− 103.4022	0.2575 (0.2175)
BMOEE	$\alpha =$ 3.8284 (0.2514) $\beta =$ 219.6406 (73.8469)	− 51.4637	− 98.9275	− 94.5482	− 97.1970	− 98.7370	0.3010 (0.1961)
UW	$\alpha =$ 6.1130 (0.5832) $\beta =$ 0.0567 (0.0197)	− 53.9658	− 103.9316	− 99.5523	− 102.2011	− 103.7411	0.2538 (0.2098)
UG	$\alpha =$ 3.4261 (0.1839) $\beta =$ 0.0040 (0.0013)	− 46.0284	− 88.0569	− 83.6776	− 86.3264	− 87.8664	0.3857 (0.0152)

### Mortality rate of COVID-19 in UK

The BEW distribution offers the best fit to the data due to the smallest AIC, BIC, CAID, AD, and highest log-likelihood value and p-value of the KS test.

### COVID-19 mortality rate in Canada

The BEW distribution offers the best fit to the data due to the smallest AIC, BIC, CAID, AD, and highest log-likelihood value and p-value of the KS test.

### COVID-19 recovery rate in Spain

The BEW distribution offers the best fit to the data due to the smallest AIC, BIC, CAID, AD, and highest log-likelihood value and p-value of the KS test.

## BEW quantile regression model

In this section BEW quantile regression model is developed using the quantile of the BEW distribution. When the response variables are bounded in the unit interval, then beta regression models indicating conditional mean responses becomes difficult to apply there. In this study, quantiles of responses are modeled using quantile regression models. Considering the quantile function of the BEW distribution, we developed the PDF for the BEW distribution.

Suppose $$\omega = {\text{Q}}\left( {y;{ }\alpha ,\lambda } \right)$$ then $${\uplambda } = \frac{{log\left[ {1{ } - { }\left( {1 - q} \right)^{{{\raise0.7ex\hbox{$1$} \!\mathord{\left/ {\vphantom {1 \alpha }}\right.\kern-0pt} \!\lower0.7ex\hbox{$\alpha $}}}} } \right]}}{{{{\beta {\text log}}}\left( \omega \right)}}$$, So, the PDF and CDF of newly developed distribution respectively, are given below.

$${\text{f}}\left( {{\text{y}};\alpha ,\lambda } \right) = {\text{~}}\alpha \beta \left[ {\frac{{log\left\{ {1{\text{~}}{-}{\text{~}}\left( {1 - q} \right)^{{{\raise0.7ex\hbox{$1$} \!\mathord{\left/ {\vphantom {1 \alpha }}\right.\kern-\nulldelimiterspace} \!\lower0.7ex\hbox{$\alpha $}}}} } \right\}}}{{\beta \log \left( \omega \right)}}} \right]y^{{\left[ {\frac{{log\left\{ {1{\text{~}}{-}{\text{~}}\left( {1 - q} \right)^{{{\raise0.7ex\hbox{$1$} \!\mathord{\left/ {\vphantom {1 \alpha }}\right.\kern-\nulldelimiterspace} \!\lower0.7ex\hbox{$\alpha $}}}} } \right\}}}{{\beta \log \left( \omega \right)}}.{\text{~}} - 1} \right]}} y^{{\left[ {\frac{{log\left\{ {1{\text{~}}{-}{\text{~}}\left( {1 - q} \right)^{{{\raise0.7ex\hbox{$1$} \!\mathord{\left/ {\vphantom {1 \alpha }}\right.\kern-\nulldelimiterspace} \!\lower0.7ex\hbox{$\alpha $}}}} } \right\}}}{{\beta \log \left( \omega \right)}}} \right]^{{\beta - 1}} }}$$

36 $$\left[ {1 - y^{{\left[ {\frac{{\log \left\{ {1~ - ~\left( {1 - q} \right)^{{{\raise0.7ex\hbox{$1$} \!\mathord{\left/ {\vphantom {1 \alpha }}\right.\kern-\nulldelimiterspace} \!\lower0.7ex\hbox{$\alpha $}}}} } \right\}}}{{\beta \log \left( \omega \right)}}} \right]^{\beta } }} } \right]^{{\alpha - 1}}$$

and

37 $${\text{F}}\left( {{\text{y}};\alpha ,\lambda } \right) = 1 - \left[ {1 - y^{{\left[ {\frac{{log\left\{ {1{\text{~}} - {\text{~}}\left( {1 - q} \right)^{{{\raise0.7ex\hbox{$1$} \!\mathord{\left/ {\vphantom {1 \alpha }}\right.\kern-\nulldelimiterspace} \!\lower0.7ex\hbox{$\alpha $}}}} } \right\}}}{{\beta {\text{log}}\left( \omega \right)}}} \right]^{\beta } }} } \right]^{\alpha }$$

here $\omega$ is the parameter of quantile. The BEW quantile is expressed as

$${\text{g}}\left( {\omega_{i} } \right) = z_{i}{\prime} \theta$$

where $z_{i}{\prime} = { }\left( {1,{ }z_{i1} ,{ }z_{i2} , \ldots ,z_{ip} { }} \right)$ are the ith covariate vectors, $\theta { } = \left( {\theta_{o} ,\theta_{1} ,{ } \ldots ,{ }\theta_{p} } \right){\prime}$ is the vectors of unknown parameters. The quantile $\in { }\left[ {0,1} \right]$ is linked to the covariates using the logit link function. So, we have

$${\text{g}}\left( {\omega_{i} } \right){ } = {\text{ logit}}\left( {\omega_{i} } \right){ } = {\text{ log}}\left( {\frac{{\omega_{i} }}{{1 - \omega_{i} }}} \right)$$

$$\omega_{i} = \frac{{exp\left( {z_{i}{\prime} \theta } \right)}}{{1 + exp\left( {z_{i}{\prime} \theta } \right)}}$$

Substitute the $\omega_{i}$ in Eq. (36) and we get

38 $${\text{f}}\left( {{\text{y}};\alpha ,\lambda } \right){ } = \alpha {\upbeta }\left[ {\frac{{log\left\{ {1{ } - { }\left( {1 - q} \right)^{{{\raise0.7ex\hbox{$1$} \!\mathord{\left/ {\vphantom {1 \alpha }}\right.\kern-0pt} \!\lower0.7ex\hbox{$\alpha $}}}} } \right\}}}{{{{\beta {\text log}}}\left( \omega \right)}}} \right]y_{i}^{{\left[ {\frac{{log\left\{ {1{ } - { }\left( {1 - q} \right)^{{{\raise0.7ex\hbox{$1$} \!\mathord{\left/ {\vphantom {1 \alpha }}\right.\kern-0pt} \!\lower0.7ex\hbox{$\alpha $}}}} } \right\}}}{{{{\beta {\text log}}}\left( \omega \right)}}.{ } - 1} \right]}} z_{i}^{\beta - 1} \left[ {1 - z_{i}^{\beta } } \right]^{\alpha - 1}$$

where $$z_{i} = y_{i}^{{\left[ {\frac{{log\left\{ {1{ } - { }\left( {1 - q} \right)^{{{\raise0.7ex\hbox{$1$} \!\mathord{\left/ {\vphantom {1 \alpha }}\right.\kern-0pt} \!\lower0.7ex\hbox{$\alpha $}}}} } \right\}}}{{{{\beta {\text log}}}\left( \omega \right)}}} \right]}}$$.

The log-likelihood for estimating the parameters bounded exponentiated Weibull quantile regression (BEWQRM) model is provided by

39 $$\ell = \mathop \sum \limits_{i = 1}^{n} log\left[ {\left( {\alpha \beta } \right)\frac{{log\left\{ {1{ }{-}{ }\left( {1 - q} \right)^{{{\raise0.7ex\hbox{$1$} \!\mathord{\left/ {\vphantom {1 \alpha }}\right.\kern-0pt} \!\lower0.7ex\hbox{$\alpha $}}}} } \right\}}}{{\beta \log \left( {\omega_{i} } \right)}}} \right] + \mathop \sum \limits_{i = 1}^{n} \left[ {\frac{{log\left\{ {1{ }{-}{ }\left( {1 - q} \right)^{{{\raise0.7ex\hbox{$1$} \!\mathord{\left/ {\vphantom {1 \alpha }}\right.\kern-0pt} \!\lower0.7ex\hbox{$\alpha $}}}} } \right\}}}{{\log \left( {\omega_{i} } \right)}} - 1} \right]log\left( {y_{i} } \right){ } + { }\left( {\beta - 1} \right)log\left( {z_{i} } \right) + \left( {\alpha - 1} \right)log\left( {1 - z_{i}^{\beta } } \right)$$

where $z_{i}$ is defined above.

The regression equations parameters are estimated by maximizing the log-likelihood (LL) function. The parameters will be written as $\hat{\user2{\alpha }}$ and $\hat{\user2{\theta }}$ of $\alpha$ and $\theta$ respectively.

The survival function and the hazard function of BEWQRM are given as

40 $${\text{S}}\left( {\text{y}} \right) = \left[ {1 - y^{{\left[ {\frac{{log\left\{ {1{\text{~}} - {\text{~}}\left( {1 - q} \right)^{{{\raise0.7ex\hbox{$1$} \!\mathord{\left/ {\vphantom {1 \alpha }}\right.\kern-\nulldelimiterspace} \!\lower0.7ex\hbox{$\alpha $}}}} } \right\}}}{{\beta {\text{log}}\left( \omega \right)}}} \right]^{\beta } }} } \right]^{\alpha }$$

and

41 $${\text{h}}\left( {\text{y}} \right){\text{~}} = \frac{{\alpha \beta \left[ {\frac{{log\left\{ {1{\text{~}} - {\text{~}}\left( {1 - q} \right)^{{{\raise0.7ex\hbox{$1$} \!\mathord{\left/ {\vphantom {1 \alpha }}\right.\kern-\nulldelimiterspace} \!\lower0.7ex\hbox{$\alpha $}}}} } \right\}}}{{\beta {\text{log}}\left( \omega \right)}}} \right]y^{{\left[ {\frac{{log\left\{ {1{\text{~}} - {\text{~}}\left( {1 - q} \right)^{{{\raise0.7ex\hbox{$1$} \!\mathord{\left/ {\vphantom {1 \alpha }}\right.\kern-\nulldelimiterspace} \!\lower0.7ex\hbox{$\alpha $}}}} } \right\}}}{{\beta {\text{log}}\left( \omega \right)}}.{\text{~}} - 1} \right]}} y^{{\left[ {\frac{{log\left\{ {1{\text{~}} - {\text{~}}\left( {1 - q} \right)^{{{\raise0.7ex\hbox{$1$} \!\mathord{\left/ {\vphantom {1 \alpha }}\right.\kern-\nulldelimiterspace} \!\lower0.7ex\hbox{$\alpha $}}}} } \right\}}}{{\beta {\text{log}}\left( \omega \right)}}} \right]^{{\beta - 1}} }} \left[ {1 - y^{{\left[ {\frac{{log\left\{ {1{\text{~}} - {\text{~}}\left( {1 - q} \right)^{{{\raise0.7ex\hbox{$1$} \!\mathord{\left/ {\vphantom {1 \alpha }}\right.\kern-\nulldelimiterspace} \!\lower0.7ex\hbox{$\alpha $}}}} } \right\}}}{{\beta {\text{log}}\left( \omega \right)}}} \right]^{\beta } }} } \right]^{{\alpha - 1}} }}{{\left[ {1 - y^{{\left[ {\frac{{log\left\{ {1{\text{~}} - {\text{~}}\left( {1 - q} \right)^{{{\raise0.7ex\hbox{$1$} \!\mathord{\left/ {\vphantom {1 \alpha }}\right.\kern-\nulldelimiterspace} \!\lower0.7ex\hbox{$\alpha $}}}} } \right\}}}{{\beta {\text{log}}\left( \omega \right)}}} \right]^{\beta } }} } \right]}}$$

**Figure 8:**
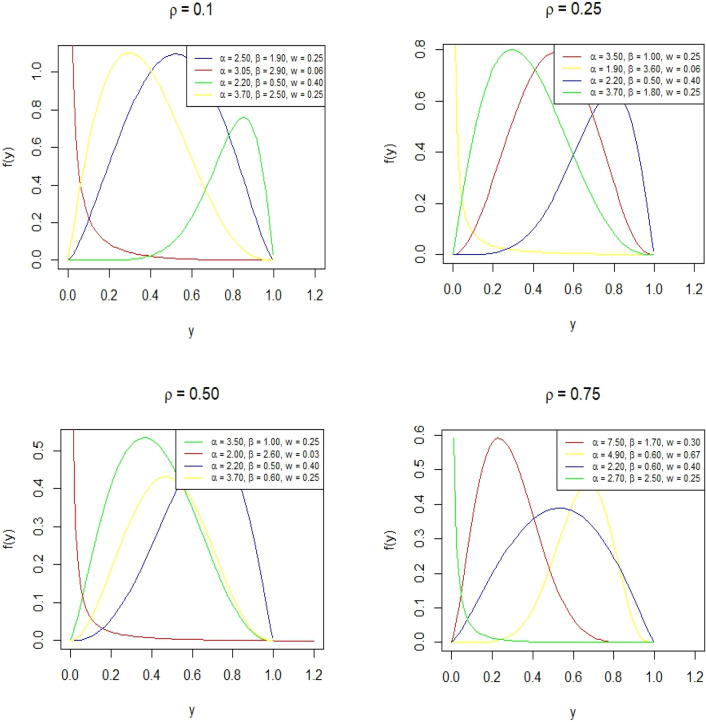
PDF plot of BEWQRM for some parametric and quantile values.

**Figure 9:**
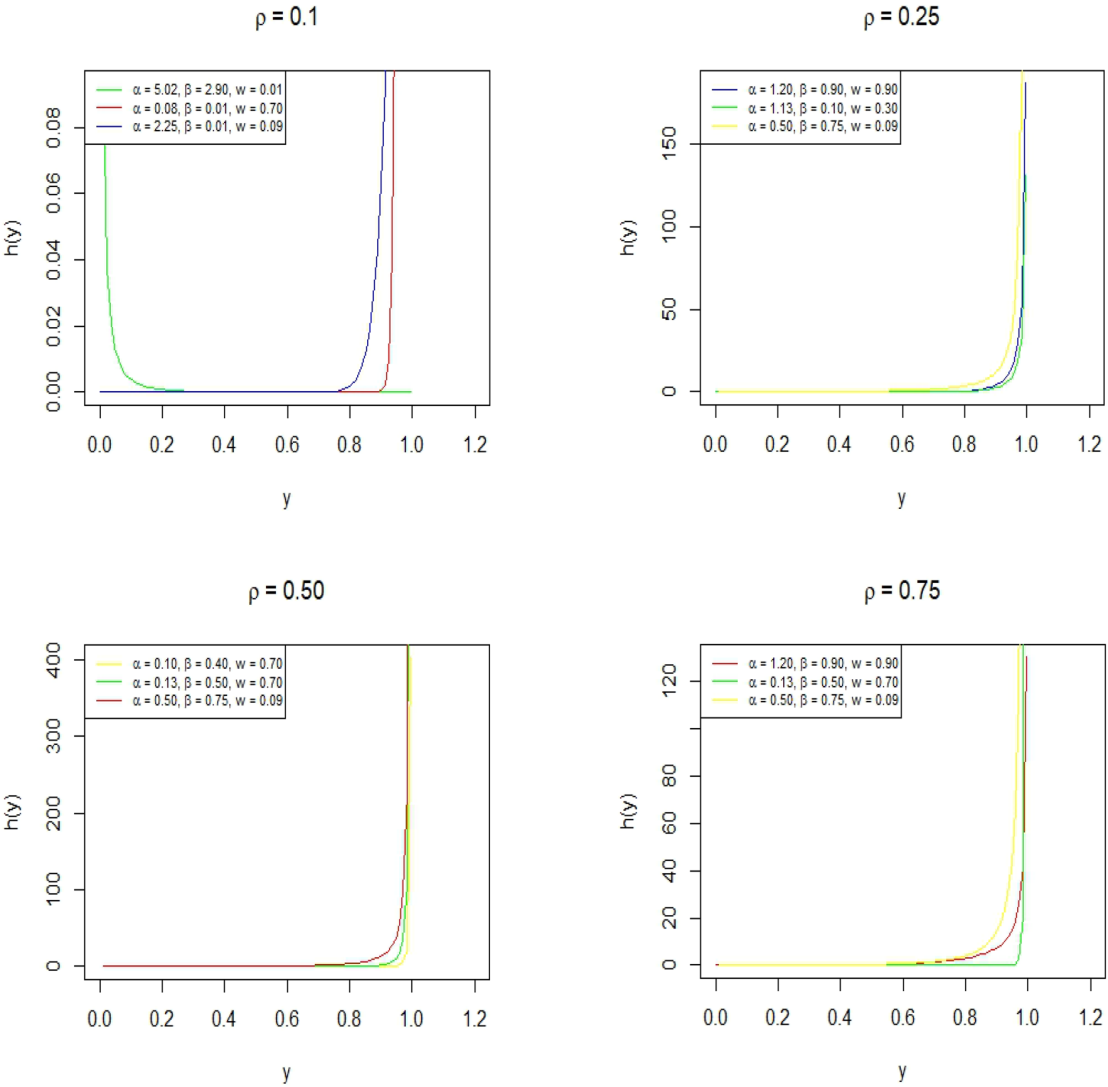
Hazard function plot of BEWQRM.

Figure 8 represents the density plot for the BEWQRM for various values of parameters. It can be seen that the PDF shows a variety of shapes such as slightly and extremely positively skewed, negatively skewed, and symmetric. Figure 9 shows the hazard rate shapes for the BEWQRM and it exhibits j shapes and reverse j shape.

## Conclusion

In various real-life situations, the random variable supports the bounded data with support [0,1] and moreover the data set shows a variety of shapes. While we model any data sets the selection of probability distribution is always a complex matter. Here in this article a new unit interval distribution is proposed named as bounded exponentiated Weibull (BEW) distribution to model data sets with support [0,1]. Although various unit interval distributions have been developed recently but firstly, every distribution is not suitable for all types of data sets, secondly, Weibull distribution has always attracted researchers due to its wide range of applications. The proposed distribution has a variety of shapes positively/negatively skewed, symmetrical, U shapes and reversed J shape. The hazard rate plot of the BEW distribution shows a bathtub shape. Various characteristics for the BEW distribution including the CDF, QF, median, moments, inequality measures, reliability measures, have been derived. Six different techniques have been investigated for estimating the parameters of the BEW distribution. A simulation has been conducted to show the performance of estimators. BEW distribution has been applied to three datasets, the data sets are COVID-19 death and recovery rates from the UK, Canada, and Spain. The proposed distribution outperforms as compared to the other competing unit interval distributions. A bivariate extension for the BEW distribution has been developed and its graphical shapes have also been shown. A BEW quantile regression model is also developed to examine the association between covariates and the conditional quantiles of unit interval response variable.

## Data Availability

The data set used and/or analyzed during the current study is available from the corresponding author on reasonable request.
